# Study protocol for testing the efficacy of the Helping Educational Leaders Mobilize Evidence (HELM) implementation strategy in elementary schools: a hybrid type 3 effectiveness-implementation randomized controlled trial

**DOI:** 10.1186/s13012-025-01429-4

**Published:** 2025-04-24

**Authors:** Jill Locke, Nathaniel J. Williams, Aksheya Sridhar, Mark G. Ehrhart, Alex Dopp, Marissa Thirion, Christine Espeland, Brandon Riddle, Kelcey Schmitz, Kurt Hatch, Lindsey Buehler, Aaron R. Lyon

**Affiliations:** 1 University of Washington, Box 357920, Seattle, WA 98115 USA; 2 Boise State University, 1910 W. University Dr, Boise, ID 83725 USA; 3 University of Central Florida, Pictor Lane, Orlando, FL USA; 4 RAND, 1776 Main Street, Santa Monica, CA 90401 USA; 5 School of Education/Educational Administration, University of Washington Tacoma, Campus, Box 358435, Tacoma, WA 98402 USA; 6 Educational Service District 105, 33 S 2nd Ave, Yakima, WA 98902 USA

**Keywords:** Schools, Implementation leadership, Implementation climate, Implementation strategy, Prevention

## Abstract

**Background:**

Schools need to implement universal student supports that prevent social, emotional, and behavioral difficulties; minimize associated risks; and promote social, emotional, and behavioral competencies. The purpose of this study is to examine the efficacy of the Helping Educational Leaders Mobilize Evidence (HELM) implementation strategy for promoting school-level implementation leadership, implementation climate, and high-fidelity delivery of an evidence-based practice. We will test HELM with an exemplar EBP, Positive Behavioral Interventions and Supports (PBIS). The specific aims of the study are to: 1) experimentally evaluate the effects of HELM versus PBIS training and technical assistance only (control condition); and 2) explore for whom, under what conditions, how equitably, and through which processes HELM works to improve outcomes, as well as its cost-effectiveness.

**Methods:**

This study will use a hybrid type 3 effectiveness-implementation trial to provide a rigorous test of the effects of HELM in elementary schools. Schools will be randomly assigned to HELM + PBIS training and technical assistance (*n* = 21 schools; n = 210 educators) or PBIS training and technical assistance only (*n* = 21 schools; n = 210 educators) in a 1:1 ratio within cohorts using covariate constrained randomization that accounts for degree of prior PBIS exposure (measured using the Tiered Fidelity Inventory at baseline) and school size. A series of mixed effects models (time within educator, educator within school) will test within-subject/between-subject interactions across three timepoints (12 months total) to examine whether HELM will show steeper gains than the control on implementation leadership (primary outcome), implementation climate, PBIS fidelity, and student outcomes. Mediational analyses will test hypothesized mechanisms of change (i.e., implementation leadership and climate) of HELM on PBIS fidelity. Sequential mixed-methods data collection and analyses will further explore how organizational mechanisms are linked to implementation outcomes. Cost-effectiveness analyses will compare costs and outcomes of PBIS training and technical assistance only versus PBIS implementation with HELM.

**Discussion:**

The nature of leadership support in schools can make the difference between successful and unsuccessful EBP implementation. Testing HELM within the context of PBIS implementation will provide rigorous evidence about whether and how HELM can equitably address important EBP and student outcomes.

**Name of the registry:**

clinicaltrials.gov.

**Trial Registration:**

Clinical Trials ID: NCT06586723. Date of Registration: August 27, 2024. Prospectively registered. URL of Trial Registry Record: https://clinicaltrials.gov/study/NCT06586723?intr=helm&rank=1

**Supplementary Information:**

The online version contains supplementary material available at 10.1186/s13012-025-01429-4.

Contributions to the Literature

- Few effective strategies to enhance evidence-based practice implementation in schools address implementation leadership and climate, and those that do have only been evaluated qualitatively.
- Schools have unique organizational characteristics, and it is important to test whether organizational implementation strategies developed in other contexts can be effective in improving implementation outcomes in schools.
- This study will evaluate the effects of a tailored adaptation of the Leadership and Organizational Change for Implementation (LOCI) strategy for schools, entitled Helping Educational Leaders Mobilize Evidence (HELM), on implementation mechanisms (implementation leadership and implementation climate), PBIS fidelity, and student outcomes.
- This study also will explore for whom, under what conditions, and how equitably HELM demonstrates its effects, providing critical information about influence on – and the limitations of – the impact of this organizational implementation strategy.
- Finally, the study will evaluate the processes through which HELM works to improve outcomes, contributing to the emerging literature on implementation mechanisms, as well as its cost-effectiveness.

## Introduction

Social, emotional, and behavioral problems occur frequently among elementary school students and dramatically impede student outcomes [1–3]. Numerous evidence-based practices (EBPs) exist to address student social, emotional, and behavioral needs, prevent problems, and ensure academic success [4]. A recent meta-analysis supports the utility of universal (i.e., “Tier 1”) interventions in improving student social, emotional, and behavioral functioning [5]. One exemplar universal EBP is Positive Behavioral Interventions and Supports (PBIS; [6–8]), a multi-tiered, problem-solving, and team-based continuum of supports that promotes all students’ social, emotional, and behavioral development [8]. Studies indicate that fidelity of PBIS delivery is highly variable across schools and often falls below levels associated with improvements in student functioning [9]. Unfortunately, variable fidelity attenuates the impact of even the most efficacious programs [10] and results that are rarely leveraged to benefit the broader population [11].

Prior research suggests that organizational factors at the level of the school building are associated with successful implementation of EBPs [12–19]. In particular, implementation leadership (i.e., proactive leader behaviors that facilitate EBP use; [20]) and implementation climate (i.e., educator perceptions of whether EBP use is valued, expected, rewarded by the school; [21]) have been repeatedly linked to higher EBP fidelity in elementary schools [13, 17, 22, 23]. Unfortunately, few effective interventions to enhance EBP implementation in schools address these factors and those that do have only been evaluated qualitatively [24].

## Helping educational leaders mobilize evidence (HELM)

To fill the gap in implementation strategies that address organizational factors within schools, our research team recently adapted the evidence-based Leadership and Organizational Change for Implementation (LOCI; [25–30]) strategy for use in the education sector. LOCI is informed by the Exploration, Preparation, Implementation, Sustainment (EPIS) framework, which details influences on implementation success over multiple phases [28]. We used a human-centered design framework [29] to enhance LOCI’s acceptability, feasibility, contextual appropriateness, usability, and effectiveness for public schools [30, 31]. The redesigned strategy – Helping Educational Leaders Mobilize Evidence (HELM) – aims to improve principals’ use of implementation leadership to support the high-fidelity delivery of EBPs that improve child outcomes [32]. HELM was designed so that it can be flexibly applied to support the implementation of any universal EBP.

**Fig. 1:**
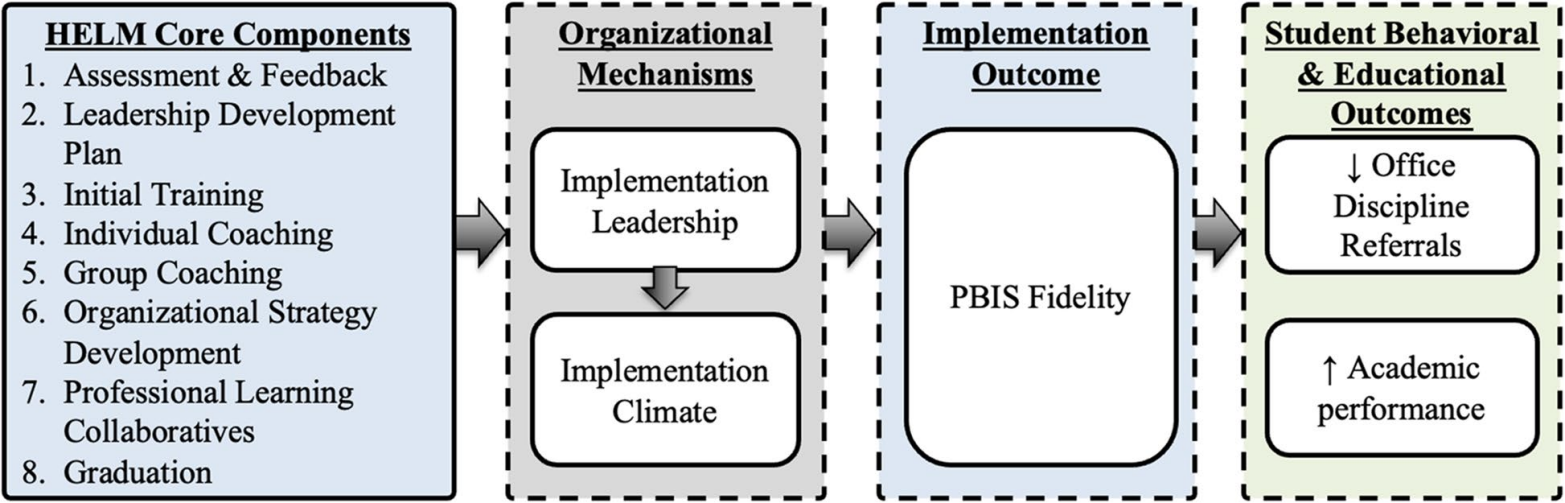
HELM Theory of Change

The HELM theory of change (Fig. 1) model depicts the HELM core components, hypothesized organizational mechanisms, implementation outcomes, and student outcomes. Identification of implementation mechanisms is critical to developing effective and streamlined implementation strategies [27–29, 33–36]. The theory of change posits that HELM will improve school implementation leadership and climate [20, 21], which leads to higher-fidelity implementation of PBIS, which then leads to improved student social, emotional, and behavioral outcomes such as disciplinary referrals [15].

## Preliminary HELM studies

Previous research found that implementation leadership and climate are malleable constructs and that changes in implementation leadership due to intervention contribute to improvements in implementation climate, EBP adoption, and observed fidelity [22, 37, 38]. Results from these studies validate key organizational mechanisms in HELM’s theory of change.

HELM was iteratively developed using the Discover, Design/Build, Test (DDBT) framework which leverages human-centered design and implementation science to guide adaptation of complex interventions for new users or settings [32]. The initial Discover and Design/Build phases involved 1) focus groups (*N* = 54 educators; 34); 2) expert input (from *N* = 15 implementation researchers and school practitioners) using a nominal group decision making process and “hackathon” solution generation (34); and 3) the Cognitive Walkthrough for Implementation Strategies (CWIS: [39]) method to evaluate HELM usability (i.e., the extent to which an intervention can be used by specified users with effectiveness, efficiency, and satisfaction; *N* = 15 principals; 30). These activities informed iterative adaptations to improve the suitability of HELM for the school context, such as aligning assessment windows to school calendars and prioritizing former school leaders as HELM coaches [31]. Furthermore, a pilot study examined the feasibility of HELM and research procedures for testing it in a large-scale Test phase trial [32]. HELM schools had significantly better implementation leadership and implementation climate (HELM’s organizational mechanisms of change) over time than control schools. Students in HELM schools also demonstrated significant increases in positive behavior compared to those in control schools, such as the extent to which their classroom teachers rated them as following directions the first time when asked (Cohen's f^2^ = 0.84, a large effect size).

## PBIS

The current project aims to test whether HELM can yield school-wide improvement in implementation outcomes and student outcomes when implementing PBIS as an exemplar EBP. PBIS organizes support across multiple tiers of intensity that vary based on the level of student need. The three tiers are: 1) Tier 1: Primary Prevention, where all students receive universal supports to increase social, emotional, and behavioral outcomes to enhance academic success; 2) Tier 2: Secondary Prevention, where some students who have not been successful with Tier 1 support alone and are at an elevated risk for problems receive supplemental support to prevent more challenging behaviors; and 3) Tier 3: Tertiary Prevention, where a few students at high risk or experiencing significant challenges receive individualized support to reduce severity (www.pbis.org). PBIS has demonstrated consistent evidence for its effects including reduced student problem behavior and improved social, emotional, and behavioral functioning in elementary schools [40–44]. PBIS is a school-wide program that integrates: 1) student and school data to identify needed supports; 2) using classroom- and student-level EBPs; and 3) implementing a systems approach designed to support fidelity. In PBIS, the system is the infrastructure (e.g., operational supports to use data) to support educators to successfully implement EBPs, and leadership is an integral part of this system.

## Study purpose and aims

The purpose of this study is to test the effectiveness of HELM on school-wide implementation of PBIS (i.e., fidelity) relative to a PBIS training and technical assistance condition. Experimental and exploratory aims are:

### Aim 1: Experimentally evaluate the effects of HELM.

Hypothesis 1a. Schools randomized to HELM will demonstrate higher PBIS fidelity compared to schools randomized to PBIS training and technical assistance only (control schools).

Hypothesis 1b. Schools randomized to HELM will demonstrate greater improvement in school context factors that support implementation (i.e., implementation leadership, implementation climate) compared to control schools.

Hypothesis 1c. Students in schools randomized to HELM will demonstrate improved social, emotional, and behavioral and academic outcomes compared to students in control schools.

### Aim 2: Explore for whom, under what conditions, how equitably, and through which processes HELM works to improve outcomes, as well as its cost-effectiveness.

Research Question 2a. Are the effects of HELM on PBIS fidelity mediated by improvement in organizational mechanisms of change (i.e., implementation leadership and climate)?

Research Question 2b. Are the effects of HELM on student outcomes mediated via implementation outcomes (i.e., PBIS fidelity)?

Research Question 2c. Are the effects of HELM consistent and equitable across buildings or are they moderated by school contextual factors (e.g., school size, student body racial/ethnic diversity, free/reduced price lunch)?

Research Question 2d. What explains implementation success in schools where HELM’s explanatory model does not fit? What factors outside of the hypothesized mechanisms explain implementation success (i.e., high fidelity) in schools where HELM’s explanatory model does fit?

Research Question 2e. What are the costs and cost-effectiveness of HELM vs. PBIS implementation as usual?

## Method

This study will use a hybrid type 3 effectiveness-implementation trial with a standard PBIS training and technical assistance only control condition to provide a rigorous test of the effects of HELM in elementary schools.

### Participants

Participating schools (*N* = 42) will be recruited into three cohorts (*n* = 14 schools p/cohort) from districts located in Washington state using a stratified recruitment approach. Schools will be compensated $400 total to support time for recruitment, retention, and other research activities. Inclusion criteria for districts are: 1) presence of a district-wide commitment to implement PBIS in elementary schools, as evidenced by either having completed a PBIS training in the past two years from certified coaches or a willingness to complete the training at the outset of study participation; 2) data sharing agreements with the project investigative team (including sharing of administrative data regarding students); 3) commitment to help recruit 10 educators per elementary school to complete study assessments (*N* = 420); and 4) no previous HELM exposure. Principals and leadership teams will participate in HELM for schools assigned to that condition. The research team will invite all educators from the school to participate in surveys and PBIS trainings and contacted by email or phone to complete informed consent. We will gather de-identified school administrative data for students in each classroom with no identifiable information and, therefore, active parental permission is not needed.

*Randomization*. Random assignment will occur at the school building level because both HELM and PBIS are building-level processes. The study methodologist will generate the randomization sequence, with allocation concealed from all other study personnel and participants. We will randomize schools within cohorts with equal probability (1:1) to: 1) PBIS + HELM; or 2) standard PBIS training and technical assistance. We will use covariate constrained randomization that accounts for degree of prior PBIS implementation and school size [45, 46]. Covariate constrained randomization enumerates a large number of possible assignments of the interventions to schools and quantifies the balance across arms with regard to a set of prespecified covariates (i.e. degree of prior PBIS implementation and school size). From a subset of possible assignments that achieve adequate balance, one is randomly chosen as the final allocation of interventions for the study.

### Implementation strategies

*HELM*. Principals, their distributed leadership teams (DLT), and school district-level leaders (e.g., Elementary Education Director, Director of Student Learning, etc.) will participate in HELM. HELM is a 9-month, data-driven organizational and leadership implementation strategy that entails eight core components: 1) Assessment and Feedback. 360º surveys measuring implementation leadership and climate are administered to principals and educators at three time points. These data will be synthesized into a detailed feedback report, which will be shared with the DLT and used to create a tailored leadership development plan to support implementation coaching throughout the year. 2) Initial Training. A 4-h didactic and interactive training will be provided to principals, their DLT, and district-level leaders and cover developing strategic implementation leadership behavior and building a positive EBP implementation climate in their schools. 3) Leadership Development Plan. During the initial training, principals and their DLT work individually with their HELM coach to review their 360º assessment data and develop goals for improving implementation leadership and climate. 4) Individual Coaching. HELM coaches provide monthly 1-h coaching sessions in person or via Zoom to review progress and update the leadership development plan. The coaching structure includes reflective questions about 1) broader school updates (10 min); 2) EBP implementation (10 min); 3) Leadership Development Plan progress (20 min); 4) barriers to implementation and solution generation (10 min); 5) next steps (5 min); and 6) “what other support is needed” from the district and/or HELM coaches (5 min). 5) Group *Coaching*. Coaches offer *optional* monthly 1-h group coaching calls with all HELM principals and DLT in each Cohort to review progress and share strategies across schools for idea generation and implementation support (this component is optional because there were mixed results in our pilot where some schools found Group Coaching helpful and others felt it was burdensome). 6) Organizational Strategy Development. Two 1-h meetings with district-level leaders are held, one in Fall semester and one in Spring semester of study enrollment to develop and update an organizational Climate Development Plan. This meeting will provide a structured discussion of alignment between school-level and district efforts to support EBP implementation. 7) Professional Learning Collaboratives. Two professional learning collaboratives are held with principals and their DLT to review content (align HELM strategies with principles from the National Educational Leadership Standards and EBP sustainment for the following school year) and share strategies across participants. 8) Graduation. During graduation, principals’ and their DLT team’s final feedback is reviewed, and progress for the past year is celebrated. Results from the pilot suggest HELM is feasible to deliver, meets the needs of school leaders, and allows school leaders to support EBP use.

*PBIS training*. Standard PBIS implementation includes initial training and technical assistance (i.e., booster training, fidelity monitoring, and coaching) – cornerstone implementation strategies across programs and domains [47] – which all participating schools will receive regardless of condition. Implementation Coaches will help schools form internal school implementation teams comprised of five to six members (e.g., teachers, administrators, community mental health providers, families). Implementation Coaches will provide ongoing support for the schools throughout the school year, inclusive of four booster training events (approximately 4–6 h) that will use progress monitoring data to determine specific skill building during the school year for all PBIS school implementation teams.

### Research procedures

Focus Groups. To understand “hypothesis defying residuals” (i.e., schools where implementation leadership and climate are inconsistent with their documented implementation outcomes), schools whose observed change in fidelity from T1 to T3 is greater than 0.5 standard deviation away from their predicted change will be identified for invitation to a focus group, balanced between users and non-users and HELM and control conditions. School-level focus groups with up to 10 participants (approximately 45–60 min) will be conducted at a convenient time for identified educators via Zoom and audio recorded. Participants will be paid $100 for their time. Recordings will be transcribed prior to coding. The mixed methods design will be sequential in structure (quantitative data collected prior to qualitative data); the functions are sampling (using quantitative data to identify our qualitative sample) and expansion (using qualitative data to provide depth and breadth of understanding of the factors that contribute to implementation outcomes that deviate from our theory of change; i.e., QUAN + QUAL); and the process is connecting (the qualitative dataset will build on the quantitative dataset; 49).

### Measures

Educators (principals/administrators, teachers, paraeducators) will complete secure web-based surveys via REDCap at three time points (Table 1). Time points will be baseline (April/May; T1) before the academic year in which schools will receive HELM, winter (Jan/Feb; T2, 8–9 months after baseline), and spring of the subsequent year (April/May; T3, 12 months after baseline). Educators will self-report their demographic characteristics, organizational mechanisms of change (implementation leadership and climate), implementation time and costs, and deidentified classroom-level outcomes. Educators will be compensated $40 for the completion of study instruments at each data collection timepoint.

**Table 1:** Study Measures

Construct	Measure Description	Source	Time
School demographics	School size, student body racial/ethnic composition, % free/reduced lunch	A	T1
	**Organizational Mechanisms**		
Implementation Leadership	*School Implementation Leadership Scale* (SILS; 15): Adapted from the original ILS [20], the SILS has 24 items loading onto 8 subscales: Proactive, Knowledgeable, Supportive, Perseverant, Communication, Vision, Available, and Distributed Leadership. Subscale internal consistencies range from 0.91 to 0.96, and scores correlate with other leadership measures	P DLT T	T1-T3
Implementation Climate	*School Implementation Climate Scale* (SICS; [48]): Adapted from the original ICS [21] includes 21 items loading onto 7 subscales: Focus on EBP, Educational Support for EBP, Recognition for EBP, Rewards for EBP, Use of Data to Support EBP, Existing Supports for EBP, and EBP Integration, with good internal consistency estimates (range: 0.81–0.90)	P DLT T	T1-T3
Burnout	*Maslach Burnout Inventory* (MBI; [49]): Burnout will be measured using the 22-item MBI. Respondents will use a 7-point Likert (“0-Never” to “6-Every Day”) to respond to items across three subscales: nine items measure emotional exhaustion (e.g., “I feel used up at the end of my workday”), five items measure depersonalization (e.g., “I feel I treat some students as if they were impersonal objects”), and eight items measure personal accomplishment (e.g., “I deal very effectively with the problems of my students”). Internal consistency was acceptable for burnout (α = .89), emotional exhaustion (α = .91), and personal accomplishment (α = .83). The lower estimate for depersonalization (α = .69) is consistent with extant research using the MBI-ES [50]	P DLT T	T1, T3
Implementation Citizenship Behavior	*Implementation Citizenship Behavior Scale (*ICBS; [51]): The School Implementation Citizenship Behavior Scale is a 12-item scale with four subscales. This study will evaluate 6 items loading onto 2 subscales: Helping Others and Keeping Informed. Research demonstrates acceptable reliability (αs = .88-.92) for all subscales	P DLT T	T1-T3
Coordination	*Team Process Scale (TPS): Coordination* [52]: Team-related processes will be measured using the 5-item Coordination response scale of the TPS. Respondents will use a 5-point Likert (“0 = not at all to 5 = to a very great extent”) to report the extent to which they perceive their team engages in effective team processes. Research supports the content and construct validity of several versions of this scale, including the 5-item Coordination response scale.	P DLT	T1-T3
Change Fatigue	*Change Fatigue* [53]: This 6-item validated measure will be used to measure the impact of organizational change on employee outcomes (e.g. well-being, withdrawal, organizational commitment, turnover intentions).	P DLT T	T1, T3
	**Implementation Outcomes**		
PBIS Fidelity	The *Tiered Fidelity Inventory* (TFI; [54]) is a 45-item tool used to provide a valid, reliable, and efficient measure of the extent to which school personnel are applying the core features of PBIS. The TFI is a group-assessment completed by a schoolwide system planning team with external facilitation with strong internal consistency (α = .96). The TFI will be collected for both PBIS + HELM and PBIS + IAU conditions across the three time points	P DLT T O	T1-T3
Implementation Cost	Costs of 1) delivering HELM to augment PBIS implementation, as well as 2) IAU, will be calculated using activity-based cost metrics. Inputs will include time, supplies, travel, overhead, and costs associated with HELM training/coaching meetings, including pre-work, scheduling, and attending meetings; as well as costs associated with PBIS training and delivery in each study condition.	A P DLT T	T1-T3
	**Behavioral and Academic Outcomes**		
Academic engagement; Prosocial behavior; & Problem behavior	*Modified Direct Behavior Rating (DBR)* will be used in which teachers observe and rate their classroom’s overall academic engagement (i.e., actively or passively participating in the classroom activity, 0–100%), disruptive behavior (i.e., interrupting activities, 0–100%), and prosocial behavior (i.e., following directions, 0–100%; Chafouleas et al., 2012). The DBR is sensitive to behavior change [55, 56] and is more feasible than assessing individual students.	T	T1-T3
Educational and Behavioral Outcomes	Standardized academic test scores, attendance rates, and disciplinary incidents (office discipline referrals, suspensions, expulsions) will be derived from administrative records	A	T1 -T3
	**HELM Manipulation Check**		
HELM Fidelity	Coding of video recordings of HELM delivery	O	T1 -T3

Note: *A* Administrative data, *P* Principal, *DLT* distributed leadership team, *T* Teacher, *O* Trained observer; T1 = Baseline (Apr/May prior to HELM/PBIS delivery year), T2 = Jan/Feb, 9 months after baseline, T3 = Apr/May, 12 months after baseline

*Fidelity*. The study’s primary outcome is school-level PBIS fidelity, assessed using the Tiered Fidelity Inventory (TFI; [54]). Facilitated by an expert PBIS coach, school PBIS implementation teams will complete the TFI in both the HELM and control conditions. The TFI is designed to be used 1) for initial assessment to determine the degree to which a school is using PBIS-consistent practices; 2) as a guide for implementation of Tiers 1, 2, and 3 EBPs, and 3) to track PBIS implementation over time. TFI data will be collected at the same time points as educator data.

*HELM fidelity checklist*. This checklist will be used as a manipulation check to document HELM delivery (based on observations of recorded HELM trainings). HELM coaches will complete a standardized measure of dates specific steps were completed.

*Qualitative Focus Groups*. We will develop a systematic, comprehensive semi-structured focus group guide that draws from the EPIS framework to examine multilevel (i.e., intervention, individual, inner, and outer settings) determinants that explain what processes facilitated or hindered implementation [28]. We will generate questions that explore the most salient implementation determinants and mechanisms, and how implementation leadership and climate may interact with other relevant characteristics of the setting [57, 58].

*Administrative Data*. At the end of each school year, deidentified academic records by classroom will be requested for all participating schools to extract students’ attendance, discipline (office disciplinary referrals), and achievement (grades, standardized test scores). To assist in modeling, these data will be collected retrospectively for the year prior to HELM participation as well as each cohort’s HELM year.

*Cost Assessments*. Activity-based costing will be used to comprehensively estimate the costs of HELM and the comparison condition [59, 60]. We will measure cost from the payor (i.e., school system) perspective, since the primary costs and associated decision-making are within the implementing school district [61]. We will isolate costs of HELM as an implementation strategy, but since HELM may have secondary impacts on PBIS costs – e.g., by increasing educator engagement in implementation – we will measure and monetize differences between study conditions in costs related to teachers’ participation in PBIS training, consultation, and delivery.

We will include open-ended items in each survey that ask about unexpected resources needed for HELM and/or PBIS. Using a sequential qual → QUANT development function, we will rapidly analyze responses on an ongoing basis so that we can immediately incorporate any newly identified cost categories into future surveys for quantitative measurement [62, 63]. To assign cost values to categories that are measured in non-monetary values (e.g., trainer time delivering HELM, teacher time spent on PBIS), we will use other data sources such as school district records or project expense reports.

### Data analysis

*Power Analysis*. The planned sample, including attrition, will provide sufficient power to test the primary hypotheses regarding the direct effects of HELM on school-, educator/classroom-, and student-level outcomes, assuming small to medium minimum detectable effect sizes of 0.23 to 0.65. These effect sizes are reasonable, likely, and clinically meaningful, based on prior research, our pilot trial, and standard interpretations of effects sizes for implementation strategies (Williams NJ, Ehrhart MG, Aarons GA, Esp S, Sklar M, Carandang K, Vega NR, Brookman-Frazee L, Marcus SC: Increasing fidelity to measurement-based care in youth mental health through improved organizational leadership and focused implementation climate: A process evaluation within a randomized trial, in preparation) [64–66]. Power analyses were conducted using Power Analysis and Sample Size Software [67]. They account for clustering of timepoints within educators/classrooms within schools (as applicable, based on the analysis), and assume final samples of 42 schools, 10–12 educators/classrooms per school (as applicable and after accounting for attrition), ICCs at the school level of 0.1 to 0.35 (depending on the outcome and as consistent with prior research), within-subjects correlations of timepoints equivalent to 50% of the variance in posttests explained by pretests (consistent with prior research), and three timepoints [68–71]. We will enroll at least 42 schools (1 extra per condition) to address potential concerns about attrition.

**Fig. 2:**
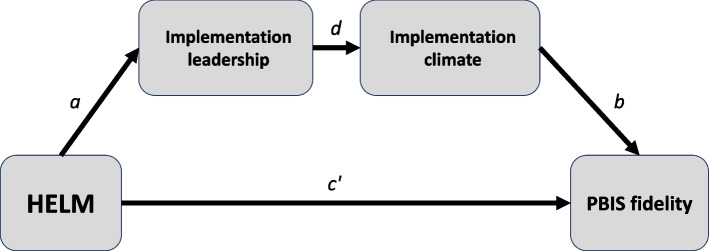
HELM mediational model: RQ2a

Anticipated statistical power for our mediation and moderation analyses vary by outcome and analytic model; however, they generally align with HELM’s anticipated effects and the effects of potential moderators based on prior research [22, 38, 72]. Making similar assumptions as above where applicable, and assuming a two-mediator, serial mediation model (Fig. 2**)**, our sample has adequate power to detect large effect sizes of 0.9 for the paths from HELM to implementation leadership and from implementation leadership to implementation climate, and a medium effect size of 0.49 for the path from implementation climate to PBIS fidelity. Minimum detectable effect sizes for analyses testing moderators of HELM’s effects on PBIS fidelity and on student outcomes range from small to medium.

*Data Analytic Approach*. We will explore for baseline equivalence between conditions on all school, teacher, and student variables following Institute of Educational Sciences guidelines, with effect sizes between 0.05 and 0.25 indicating that statistical adjustment for nonequivalent baseline characteristics is required [73]. Non-equivalent baseline characteristics will be included as covariates at the school, educator, or student level, as appropriate. Following best practice guidelines, variables used for covariate constrained randomization will be included as covariates in all models [46, 74]. Data missing at random will be modeled using full information maximum likelihood estimation in mixed effects modeling or multiple imputation for other analyses. Although all analyses will use an intent-to-treat approach, we will examine the robustness of condition assignment through descriptive analyses of scores on the HELM fidelity tools. Schools that achieve 80% or higher of the maximum possible score on all HELM fidelity criteria will be considered having received a full dose of HELM training and coaching.

All analyses will use an intent-to-treat approach in which units are analyzed based on condition assignment regardless of HELM implementation success [75]. Because the trial includes outcomes at multiple levels, the specific analytic approach will vary depending on the outcome. However, in general, analyses will employ 2- or 3-level mixed effects models reflecting data collection time points nested within educator/classroom nested within schools. We will test for significantly large ICCs at the district levels to determine if statistical nesting is necessary. Standard model-building procedures will be used [76, 77], including fitting a null model to facilitate calculation of variance accounted for in later models, fitting unconditional growth models (e.g., linear, quadratic, piecewise) to determine the optimal functional form for time, and finally, fitting models that include the variable for condition (and the condition by time interaction, as appropriate) along with covariates. Iterative models with possible covariates will be tested. Covariates not significantly contributing to the model at *p* < 0.10 based on likelihood ratio tests will be removed. We will obtain estimates of whether there were statistically significant differences between conditions on rate of change over time (i.e., slope), and whether there are statistically significant condition differences in average score on each outcome at T2 and T3. Models will be generalized, with appropriate link functions (e.g., log-link, Poisson) applied based on distributional form (e.g., dichotomous, zero-inflation). Estimations will be fit using full maximum likelihood. Models will be assessed for possible violations of assumptions. Inference will be evaluated relative to *p* < 0.05. For hypotheses/research questions with multiple DVs, we will adjust for familywise error using Benjamini and Hochberg’s False Discovery Rate [78].

*Qualitative Analysis.* Certain codes will be conceptualized during the protocol guide development and driven by the EPIS framework (i.e., deductive approach) and others will be developed through reading an initial subset of transcripts (i.e., inductive approach). Themes will provide a way of understanding the most salient factors that impact implementation and extend beyond the existing HELM organizational mechanisms and theory of change [79, 80]. EPIS-driven directed coding will include system levels (i.e., intervention, individual, inner setting, outer setting) as initial “parent nodes.” After a stable set of codes is developed, a consensus process will be used in which all reviewers independently code and compare their coding to arrive at consensus judgments through open dialogue [81–83].

*Cost and Cost-Effectiveness Analyses*. We will use the CostOut program to complete the cost analysis for HELM and PBIS implementation-as-usual. CostOut specifies ingredients for each intervention condition, assigns prices (national and user-inputted local values), and calculates costs based on the units per ingredient used [59, 84]. We will use CostOut to generate descriptive statistics describing typical costs (i.e., means, standard deviations) for HELM and PBIS. We will calculate total costs for each condition, and incremental costs of HELM over PBIS training and technical assistance only. We also will provide cost breakdowns to help administrators understand the budget implications of HELM, including personnel versus other direct expenses and start-up versus maintenance costs. Finally, we will conduct sensitivity analyses to examine the robustness of our cost estimates by identifying areas of uncertainty in measuring the units and prices for our ingredients, and then calculating costs across a range of plausible values [85, 86].

We also will use CostOut to calculate the cost-effectiveness of HELM versus PBIS [87]. This will involve calculating a series of incremental cost-effectiveness ratios for each student outcome (social, emotional, behavioral, academics) and PBIS implementation outcome (fidelity). Finally, we will again use sensitivity analyses to examine the robustness of our cost-effectiveness results, both across the ranges of costs examined and across plausible effectiveness estimates (i.e., 95% CIs for student and implementation outcomes).

## Discussion

One out of 5 elementary students exhibit social, emotional, and behavioral difficulties that hinder their academic success which are linked to later negative outcomes, including substance use problems, unemployment, houselessness, and contact with the legal system [1, 88, 89]. Despite the promise of EBPs like PBIS to promote student social, emotional, and behavioral functioning, their routine use in schools is limited, reducing their large-scale impact on student outcomes [90, 91]. The majority of schools in the United States are attempting to implement multi-tiered frameworks such as PBIS, yet evidence suggests that implementation in educational settings is typically absent, inconsistent, or incomplete [91, 92]. School leadership plays a pivotal role in the successful implementation of EBPs that effectively reduce social, emotional, and behavioral outcomes [13, 93]. The presence or absence of strong leadership support in schools can make the difference between successful implementation and abandonment. However, leadership is not specifically targeted in standard PBIS training [93].

Research on leadership-focused implementation strategies have almost exclusively focused on outpatient mental health clinics or clinics focused on treating substance use disorder [25–27, 27–31] and has not been widely evaluated in public schools. There is some preliminary evidence to suggest HELM positively impacts implementation leadership, implementation climate, and implementation citizenship and may potentially buffer the decline in EBP implementation efforts that naturally occurs over the school year [94]. This study will be the first full-scale test of HELM’s efficacy in public schools, which is a very different service setting with unique organizational characteristics, systems, and processes. It is important to test whether the type of leadership-focused training and climate development that worked in outpatient mental health clinics or clinics focused on treating substance use disorder also can work in public schools.

HELM has the potential to reduce the substantial waste in time and resources resulting from ineffective universal EBP implementation, including inadequate uptake and low fidelity. HELM is closely aligned with the needs and priorities of educators working in educational settings due to its applied focus and emphasis on strategic implementation behaviors. Demonstrating the effectiveness of HELM on PBIS fidelity will address a highly prevalent barrier to improved population health: abandonment or low-fidelity delivery of effective interventions focusing on social, emotional, and behavioral functioning in schools. Because HELM was developed and tested with school partners, it has the potential to be highly usable and scalable, which has significant implications for low-resource community contexts in which lay providers deliver services. A unique aspect of HELM is that it emphasizes the engagement of DLT in schools, which is a common leadership model in schools but not in outpatient mental health clinics or clinics focused on treating substance use disorder. As of February 2025, no participants have been enrolled.

### Limitations

We recognize that it will be difficult to identify school districts that have not engaged in some level of PBIS implementation. However, for maximum generalizability and educational impact, HELM needs to be able to support both initial implementation of an EBP as well as improve implementation efforts that are already in process. Schools with variable levels of PBIS implementation will allow us to examine this effect.

## Supplementary Information

Supplementary Material 1.

## Data Availability

The application described in this manuscript is freely available. Please contact the lead author for more information.

## Abbreviations

CWIS: Cognitive Walkthrough for Implementation Strategies
DBR: Direct Behavior Ratings
DDBT: Discover, Design/Build, Test
DLT: Distributed Leadership Teams
EBP: Evidence-based practice
EPIS: Exploration, Preparation, Implementation, Sustainment
ES: Effect Sizes
HELM: Helping Educational Leaders Mobilize Evidence
ICBS: Implementation Citizenship Behavior Scale
IES: Institute of Education Sciences
LOCI: Leadership and Organizational Change for Implementation
MBI-ES: Maslach Burnout Inventory – Educators Survey
MDES: Minimum Detectable Effect Sizes
ML-PA: Multilevel path analysis
MLR: Maximum likelihood estimator
PASS: Power Analysis and Sample Size Software
PBIS: Positive Behavioral Interventions and Supports
REDCap: Research Electronic Data Capture
TFI: Tiered Fidelity Inventory
TPS: Team Process Scale
